# Studies towards synthesis, evolution and alignment characteristics of dense, millimeter long multiwalled carbon nanotube arrays

**DOI:** 10.3762/bjnano.2.34

**Published:** 2011-06-14

**Authors:** Pitamber Mahanandia, Jörg J Schneider, Martin Engel, Bernd Stühn, Somanahalli V Subramanyam, Karuna Kar Nanda

**Affiliations:** 1 Leibniz Institut für Polymerforschung e.V., 01069 Dresden, Hohe Strasse 6, Germany,+49-351-4658-639; 2 Fachbereich Chemie, Eduard-Zintl-Institut, Anorganische Chemie, Petersenstr. 18, Technische Universität Darmstadt, D-64287 Darmstadt, Germany; 3 Department of Solid State Physics, Technische Universität Darmstadt, Hochschulstr. 6, 64289 Darmstadt, Germany; 4 Department of Physics, Indian Institute of Science, Bangalore-560012, India; 5 Materials Research Centre, Indian Institute of Science Bangalore-560012, India

**Keywords:** carbon nanotubes, characterization, synthesis

## Abstract

We report the synthesis of aligned arrays of millimeter long carbon nanotubes (CNTs), from benzene and ferrocene as the molecular precursor and catalyst respectively, by a one-step chemical vapor deposition technique. The length of the grown CNTs depends on the reaction temperature and increases from ~85 µm to ~1.4 mm when the synthesis temperature is raised from 650 to 1100 °C, while the tube diameter is almost independent of the preparation temperature and is ~80 nm. The parallel arrangement of the CNTs, as well as their tube diameter can be verified spectroscopically by small angle X-ray scattering (SAXS) studies. Based on electron diffraction scattering (EDS) studies of the top and the base of the CNT films, a root growth process can be deduced.

## Introduction

CNTs have been extensively studied in recent years due to their unique structural and physical features, and chemical and mechanical properties [1] as well as for their potential technological applications. In particular, vertically aligned, long CNTs with high density are promising for device applications that require a large effective surface area and a high aspect ratio, such as, e.g., field electron emitters [2], gas storage media [3], or chemical sensors [4]. Thus, several approaches have been undertaken to obtain long, aligned CNTs over the last decade or so [5–22]. Additional reasons for these efforts stem from the ongoing challenge to integrate CNTs into micro components, which requires covering large areas in an ordered fashion, in order to implement them in higher integrated device architectures [23]. Such a massive parallel arrangement also requires reliable connection of such CNT arrays in order to use them in, e.g., micro scaled electronic devices. Recently, we have shown that making electrical contact to CNT arrays is indeed possible if such CNT arrays are synthesized over larger substrate areas and display sufficient mechanical stability [24].

Concerning the CNT synthesis, growth promoters such as thiophene, pure sulfur or hydrogen, and chemical oxidants such as oxygen, organic molecules (e.g., ethanol, ethers, aldehydes, ketones) or water have been employed for preparing ultra-long, aligned CNTs [8,23–25]. The ratio of the carbonaceous C*_x_*H*_y_* molecular source used and additional growth promoters such as H_2_, as well as H_2_O or oxygen containing organic molecules such as ethers, or ketones as oxidizing agents, have a subtle influence on the purity, growth rate and final growth height of vertically oriented CNTs. Oxygen containing organic molecules and water act as oxidizers and thereby increase the activity and lifetime of the metallic catalyst particles by removing amorphous carbon, which is typically formed during the growth process, from the particle’s surface. Due to this undesired deposition of carbon, the catalyst becomes poisoned in the early stages of the CNT growth. Nevertheless, there are reports on the vertical growth of up to mm long CNTs, even without the additional supply of such oxidizing agents [13,26–28]. Thus, the exact role of such promoters and oxidizers is still unclear. Despite the intensive studies towards this end, it remains an interesting task to provide a straightforward and low-cost production method for obtaining good quality aligned multi-walled CNTs (MWCNTs) by a straightforward and easy to use technique.

Herein we report on the synthesis of ultra long, aligned, MWCNTs using benzene and ferrocene as molecular precursor and catalyst respectively, employing a single step, atmospheric pressure, CVD technique, which requires no additional carrier (e.g., Ar) or process gas (e.g., H_2_), and no oxygen containing compound as oxidant, for growing mm long CNT arrays. In addition, we show that SAXS is a valuable technique for studying the alignment of CNTs up to μm sized dimensions.

## Results and Discussion

**Figure 1:**
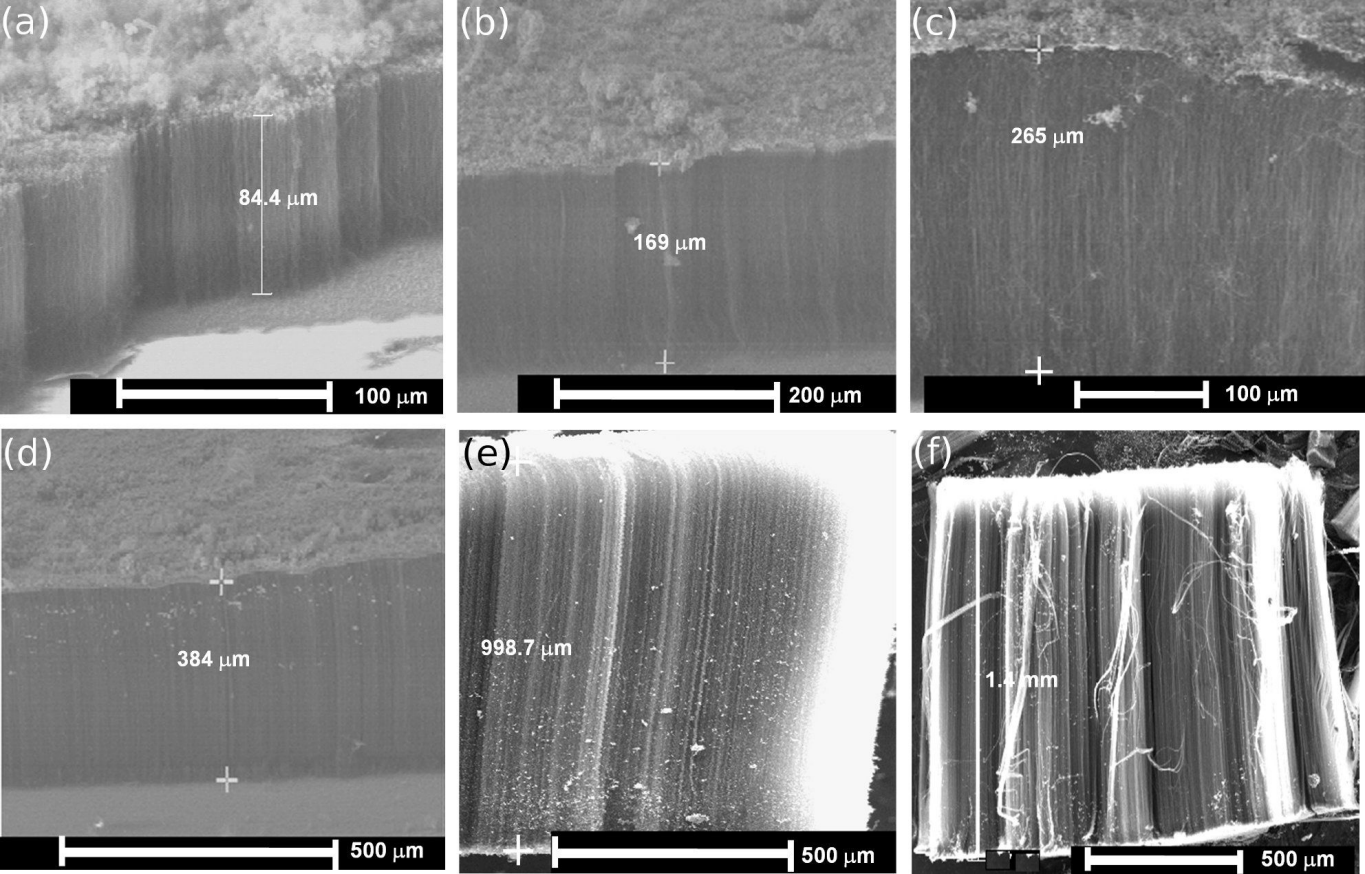
SEM images of aligned CNTs prepared at different temperatures (a) 650 °C, (b) 750 °C, (c) 850 °C, (d) 950 °C, (e) 1050 °C, and (f) 1100 °C.

**Figure 2:**
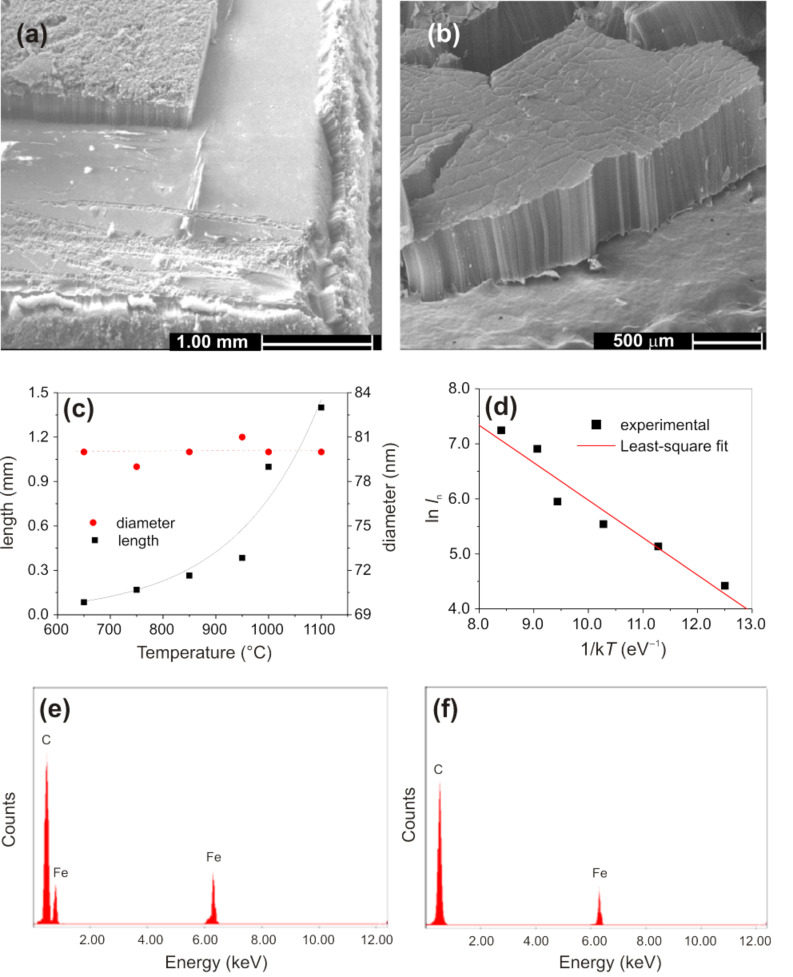
(a) SEM image of aligned CNTs on a quartz substrate. (b) SEM image of an isolated mat collected from the inner wall of the quartz tube. (c) The length and diameter of CNTs as a function of the preparation temperature; (d) shows the length (*l*_n_) Arrhenius plot. (e) EDS pattern taken from the quartz surface after the mat is peeled off and (f) from the top of the aligned CNTs structure.

Scanning electron microscope (SEM) images of aligned CNTs, prepared in the temperature range of 650 to 1100 °C, with ferrocene/benzene as catalyst and precursor, are shown in Figure 1. The length of the grown CNTs increased with temperature, and up to millimeter long CNTs were obtained at a temperature of 1100 °C. Furthermore, the formation of a significant amount of amorphous carbon was found on the top of the grown CNTs when the synthesis temperatures did not exceed 650 °C. However, on increasing the temperature to 1100 °C, the deposition of amorphous carbon was significantly reduced under the same reaction conditions, i.e., the same precursor gas composition, and the formation of CNTs was highly favorable. A CNT felt-like material (CNT mat) containing vertically aligned CNTs could then be routinely collected from the inner walls of the quartz reaction tube or from a quartz substrate placed within (Figure 2b). The length of the as-synthesized CNTs as a function of the synthesis temperature increased monotonically (Figure 2c).

**Figure 3:**
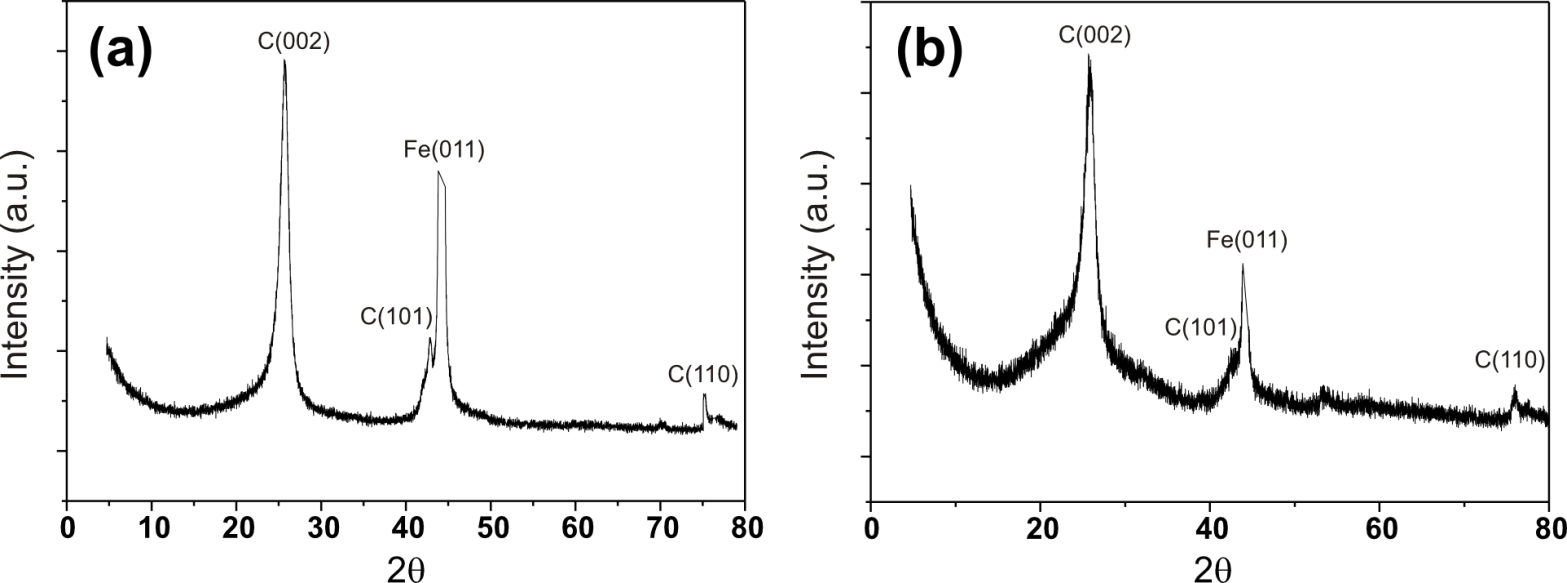
X-ray diffraction (XRD) of aligned MWCNTs on quartz substrate prepared at (a) 650 °C and (b) 1100 °C. The XRD was performed without peeling off aligned MWCNTs from the surface of the quartz substrate.

An Arrhenius plot (Figure 2d) showing the length (*l*_n_) dependence of the CNTs on the furnace temperature allows us to calculate the activation energy for the CVD synthesis process and was found to be 0.68 eV [29]. It is noteworthy that the activation energy (*E*_A_) was reported to be 1.2–1.8 eV for a thermally activated CVD process and significantly lower, at ~0.3 eV, for a plasma enhanced CVD process (PECVD) [30]. A higher activation energy indicates that the growth of CNTs is mainly by bulk diffusion, while a lower activation energy is due to a surface diffusion limited process. The calculated value of *E*_A_ in the CVD process employed herein, suggests that the increase in length is mainly due to an enhanced surface diffusion process of the reactive carbon species with increasing synthesis temperature. The alignment of the CNTs can be attributed to the formation of a high density of catalytically active iron particles, which allows for a very dense and simultaneous vertical growth of CNTs. Once the initial CNT formation is established, CNT growth continues in the vertical direction and is further reinforced by the presence of nearby surrounding CNTs, which display multiple van der Waals force interactions and mechanically stabilize the large area growth in the vertical direction [31]. The fate of the iron catalyst in the CNT array structure can be probed by EDS. The elemental composition of the surface of the quartz substrate after the aligned mat was peeled off (Figure 2e), and on the top of the aligned CNTs (Figure 2f), reveals that the iron content on the quartz surface was much higher (Figure 2e) compared to that on top (Figure 2f). This finding suggests a strong interaction between support and catalyst and points to a base growth process during CNT formation [32–34]. X-ray diffraction (XRD, Cu Kα, λ = 1.5406 Å) measurements were performed on materials grown at 650 and 1100 °C on the quartz substrate without peeling off the aligned MWCNTs, and the results are shown in Figure 3. The XRD results confirm the graphitic nature of the MWCNTs and the presence of the Fe catalyst.

**Figure 4:**
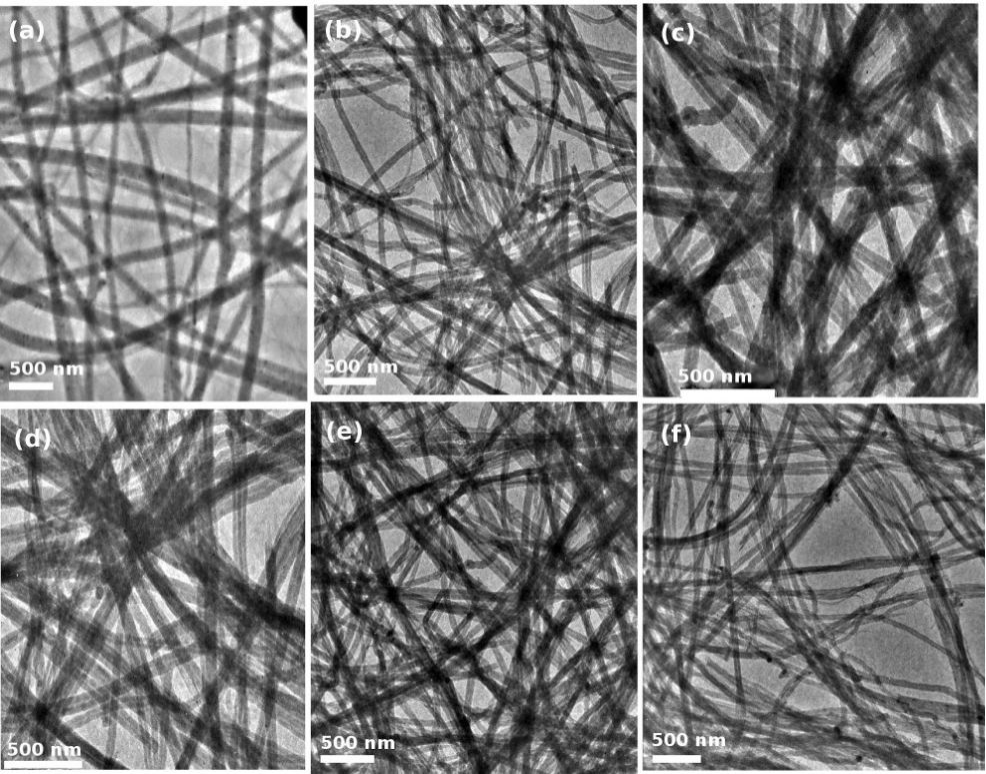
TEM images of aligned CNTs prepared at (a) 650 °C, (b) 750 °C, (c) 850 °C, (d) 950 °C, (e) 1000 °C, and (f) 1100 °C. The diameter of CNTs is almost independent of the synthesis temperature.

**Figure 5:**
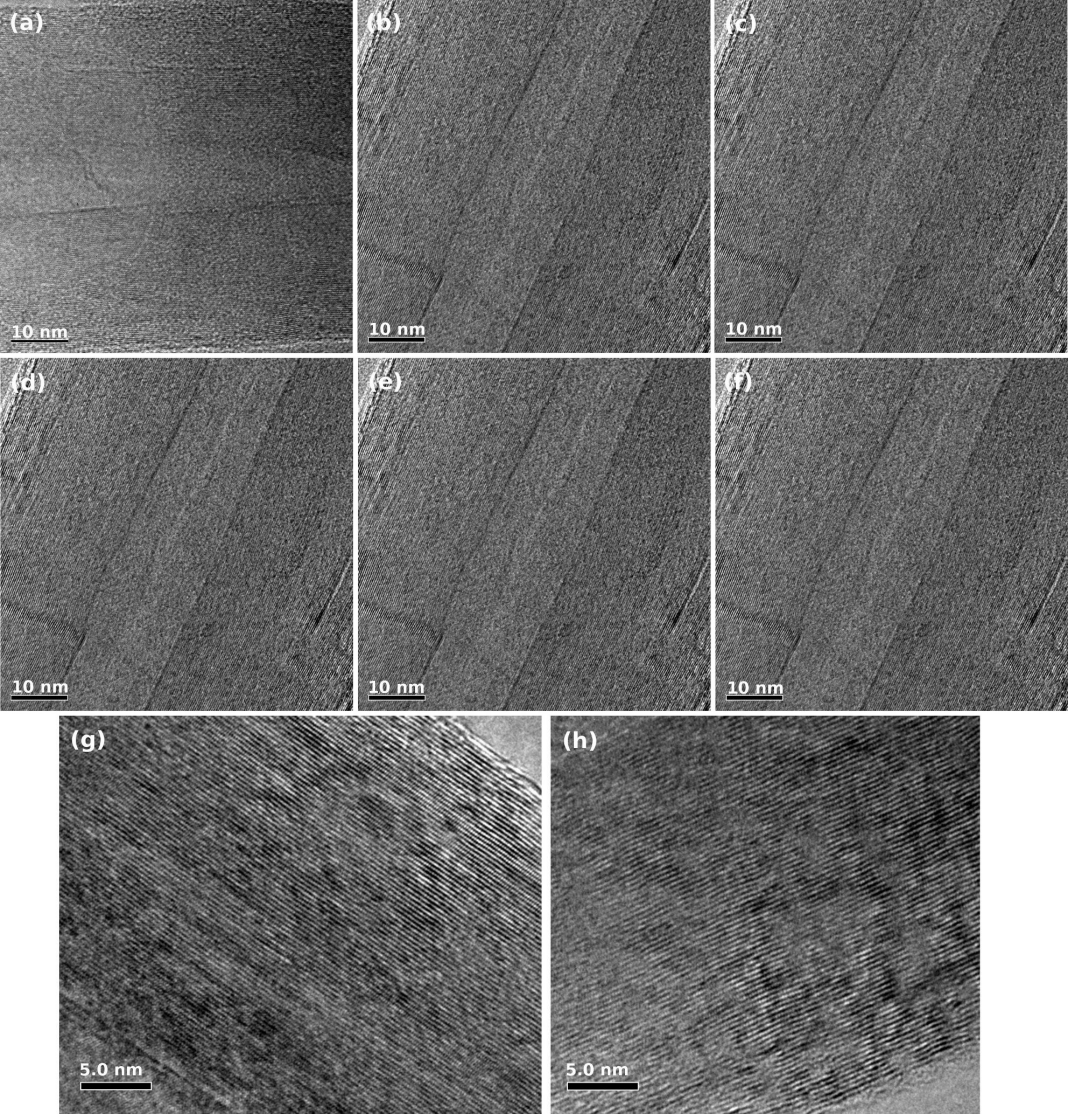
HRTEM images of MWCNTs prepared at (a) 650 °C, (b) 750 °C, (c) 850 °C, (d) 950 °C, (e) 1000 °C, and (f) 1100 °C. Representative HRTEM images of the tube walls of MWCNT synthesized at (g) 650 °C and (h) 1100 °C.

The microstructure of the aligned CNTs was investigated using TEM (Figure 4). It is interesting to note from Figure 2c that the diameter was almost independent of the preparation temperature. High resolution TEM (HRTEM) images (Figure 5a–f) of CNTs, prepared in the temperature range between 650–1100 °C, confirm the well crystallized multi-layered graphitic tubular structure with more than 50 layers and outer diameters of 75–85 nm (Figure 5g and 5h). HRTEM micrographs taken on one side of a MWCNT prepared at 650 and 1100 °C reveal that the CNTs are well crystallized. No catalyst particles were found either inside the tubes or embedded in the wall structure of the CNTs.

It has been well established that the diameter of the CNTs formed in a CVD process depends strongly on the size of the catalyst particles used [35]. In a typical CVD process, either the catalyst and carbon source materials are continuously supplied, or only the carbon source is supplied during the process and the catalyst particles are pre-deposited on the substrate, e.g., by vapor deposition or even by ink jet printing [36–37]. Typically, at elevated synthesis temperatures, larger catalytic particles are formed due to the increasing mobility of the as-deposited catalyst particles on the substrate surface compared to lower temperatures [38]. The continuous supply during the deposition process of the source materials also contributes to the increase of the CNT diameter. Consequently, CNTs of larger diameter are obtained at higher synthesis temperatures [35–40]. In the present synthesis methodology, the experimental set up was modified such that the precursor mixture vaporized and the gas-phase precursor species were driven to the hot reaction zone without any additional transport gas. In the hot reaction zone, the precursor mixture pyrolyzed into carbon species and iron, resulting in the formation of CNTs on the quartz substrate or the inner walls of the quartz tube. Due to the chosen set up, there was no steady and continuous supply of catalyst or carbon source material to the reaction zone. Thus, the catalyst particle concentration remained nearly constant and low, such that during the growth of the CNTs no obvious, significant agglomeration of catalyst particles, to form larger aggregates beyond a certain size, occurred. This resulted in only a minor increase of the CNT diameter, even when the synthesis temperature was increased over a wide range (650–1100 °C).

**Figure 6:**
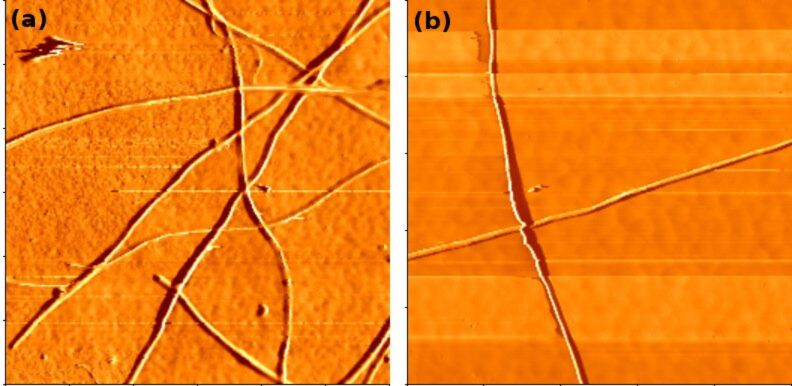
AFM image of isolated CNTs prepared at (a) 650 °C and (b) 1100 °C. The scan area is 15 μm × 15 μm and 20 μm × 20 μm, respectively. The isolated CNTs are mechanically detached from the CNT block structures.

**Figure 7:**
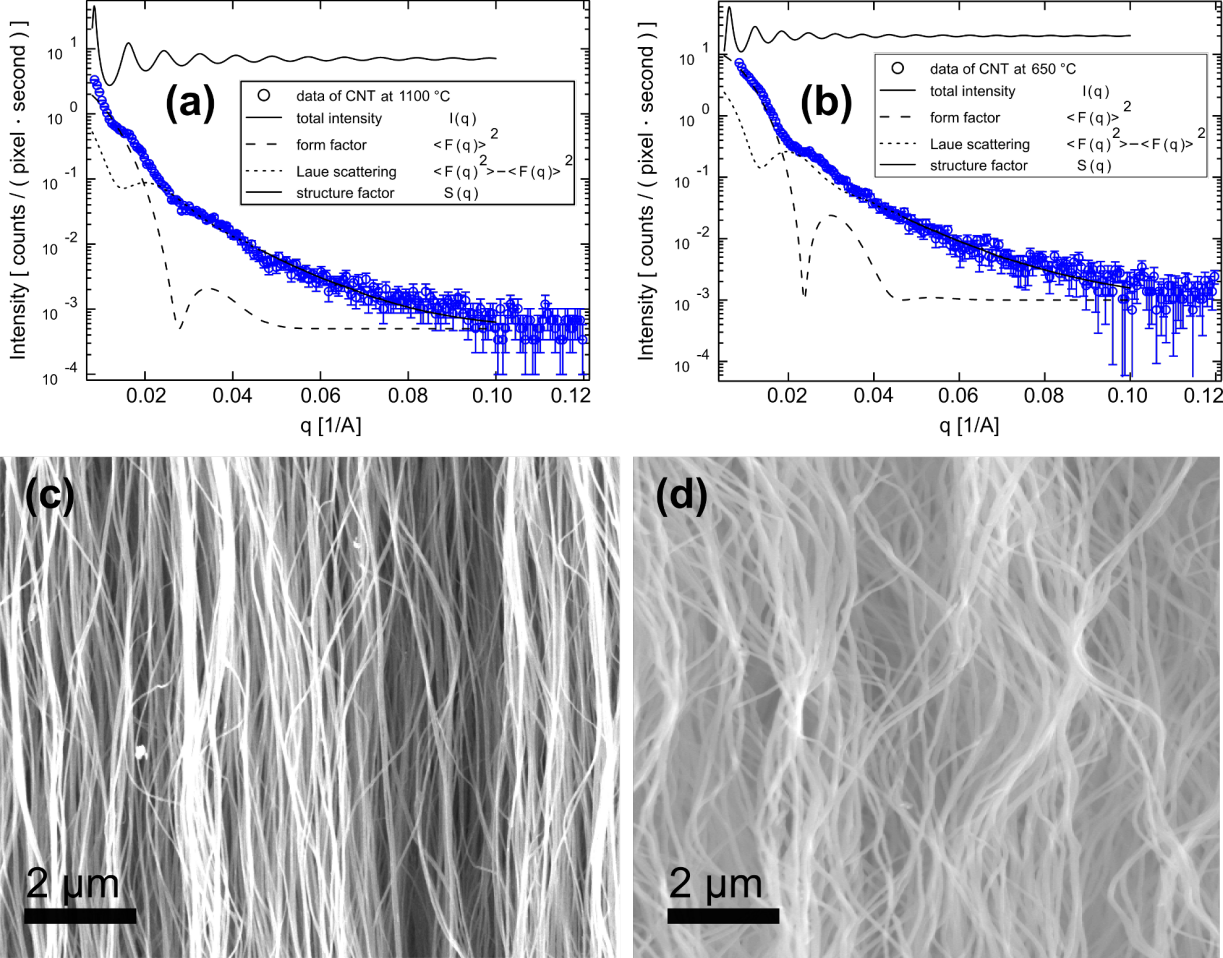
SAXS pattern of aligned CNTs prepared at (a) 1100 °C and (b) 650 °C. SEM images of aligned CNTs prepared at (c) 1100 °C and (d) 650 °C.

Single and isolated long CNTs can be detached easily from the aligned CNT bundle or mat like structure. Atomic force microscope (AFM) images of individual CNTs, synthesized at 650 °C and 1100 °C, are depicted in Figure 6. They confirm the uniform diameter as found by TEM. SAXS and small angle neutron scattering (SANS) are powerful indirect techniques to study the orientation of aligned CNTs and to provide information on their average diameters [41–47]. Accordingly, values for CNT diameter were extracted from the SAXS patterns. The observed SAXS intensity can be described as


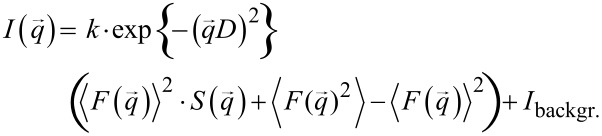


where 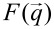 is the form factor of the cylindrical rod given as 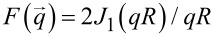 with the tube radius *R*, the scattering wave vector *q* = 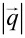 and *J*_1_ is the first order Bessel function [48]. 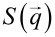 describes the structure, i.e., the arrangement of the pores, *D* corresponds to the roughness at the tube interfaces, *k* is a scaling factor due to arbitrary units of intensity and *I*_backgr._ is the constant background intensity. The brackets denote the average, with respect to the radius, of the cylinders modelled with a Schulz–Zimm distribution. The intensity curve was extracted from the two-dimensional (2D) detector with a narrow rectangular filter [49]. The structure was modelled according to a calculated structure factor [49–50]. Figure 7a and 7b present the resulting scattering intensity and the contribution of single terms from the scattering experiment. A diameter of ~80 nm, obtained from the best fit of the SAXS experiment, is indeed comparable with the results from the HRTEM studies for individual CNTs samples, e.g., prepared at 1100 °C (Figure 7a). However, a diameter of 100 nm was determined for CNTs prepared at a significantly lower synthesis temperature of 650 °C (Figure 7b). Therefore the diameter for these CNTs obtained at lower temperatures was seen to be significantly bigger (~100 nm) than that obtained from the HRTEM studies (~80 nm). This discrepancy is probably due to the larger inter-tube distance for CNTs prepared at the lower temperature. Indeed it can be inferred from SEM that the aligned CNTs obtained at 1100 °C (Figure 7c) show a narrower inter-tube distance with a more dense packing than those prepared at 650 °C (Figure 7d) which are more loosely packed.

**Figure 8:**
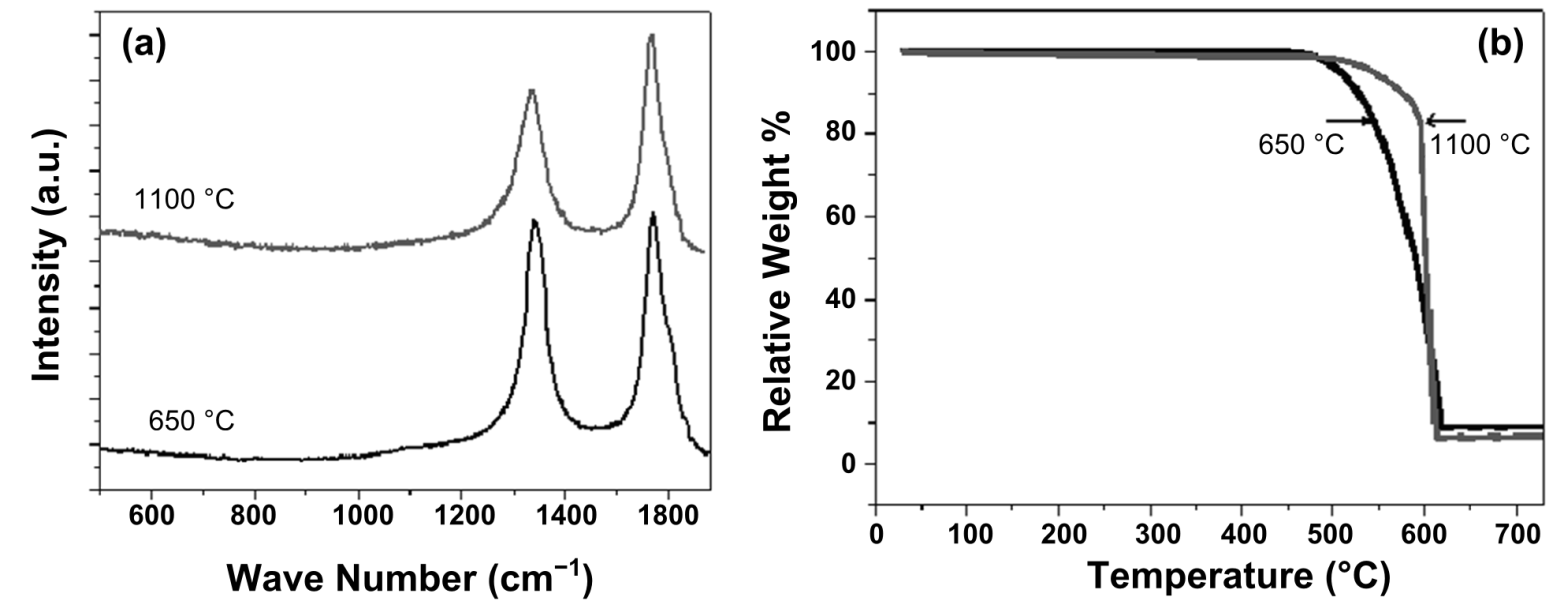
(a) Raman spectra and (b) TGA curves of aligned CNTs prepared at 650 and 1100 °C.

Figure 8a shows the Raman spectra of the aligned CNTs prepared at the lowest synthesis temperature of 650 °C, as well as the highest temperature of 1100 °C. Two peaks at 1348 and 1578 cm^−1^ were observed. The tangential mode at 1578 cm^−1^ (G band) corresponds to crystalline graphitic layers, while the peak at 1348 cm^−1^ (D band) corresponds to the disordered, amorphous fraction of the CNT sample. The intensity ratio *I*_D_/*I*_G_ gives information on the amount of non-graphitic (sp^3^) versus graphitic (sp^2^) carbon species [51–52]. The ratio *I*_D_/*I*_G_ was found to be 0.91 and 0.583 for samples prepared at 650 and 1100 °C, respectively. The latter reduced value of *I*_D_/*I*_G_ corresponds to a higher degree of graphitisation in CNT formation and a lower sp^3^ carbon content (due to residual non-graphitic carbon) in the samples prepared at 1100 °C.

Thermogravimetric analysis (TGA) measurements were carried out for the synthesized CNT material grown at 650 and 1100 °C in a dry air atmosphere with a heating rate of 10 °C/min and show the typical temperature behavior of high quality CNTs (Figure 8b). The weight loss for CNTs synthesized at 650 °C, started at 490 °C and continued to increase rapidly with temperature, until a stable plateau at 620 to 750 °C (final point of measurement) was reached, leaving a residual weight of 8 wt %. The weight loss for CNTs synthesized at 1100 °C started at 525 °C, until a stable plateau was reached at 715 to 750 °C, leaving a residual weight of ~7 wt %. Thus a purity of between about 92 and 93% of the total mass can be estimated. The remaining masses in both samples may come from residual graphitic carbon impurities which decompose at significantly higher temperatures and from the remaining iron catalyst.

## Conclusion

In conclusion, we report the growth of millimeter-long CNTs by a direct CVD method. The synthesis technique is a one-step CVD process in which no carrier gas, pre-deposited metal catalyst particles, or growth promoters such as oxidants are required for the preparation of long, vertically aligned CNTs. It was shown that the length of CNTs increased with increasing synthesis temperature, while their diameter was almost independent of the synthesis temperature over a wide range. A base growth mechanism of aligned CNTs, supported by the experimental results, was proposed. It could be shown that the SAXS technique offers a versatile spectroscopic tool for determining the CNT diameter as well as the CNT alignment in a bundle arrangement.

## Experimental

A mixture of ferrocene (~18 mg) and benzene (2mL) was placed in quartz tube of internal diameter 1.0 cm and length 70 cm, closed at one end [53–55]. The other end of the quartz tube was connected to a rubber bladder to collect the exhaust gases. The precursors were vaporized inside a horizontal furnace and heated to the desired pyrolysis temperature (range 650–1100 °C) at a rate of 20 °C/min. When the final reaction temperature was reached, it was maintained for 30 minutes and the reactor was then cooled to room temperature. The growth of aligned CNTs occurs at the inner walls of the quartz tube in the hot reaction zone. The isolated material was characterized by SEM, transmission electron microscopy TEM, AFM, EDS, TGA and Raman spectroscopy.

## References

[1] Dresselhaus M S, Dresselhaus G, Saito R (1995). Carbon.

[2] Yilmazoglu O, Popp A, Pavlidis D, Schneider J J J (2010). Vac Sci Technol, B: Microelectron Nanometer Struct-Process, Meas, Phenom.

[3] Gao H, Wu X B, Li J T, Wu G T, Lin J Y, Wu K, Xu D S (2003). Appl Phys Lett.

[4] Modi A, Koratkar N, Lass E, Wei B, Ajayan P M (2003). Nature.

[5] Lee C J, Lyu S C, Kim H-W, Park C-Y, Yang C-W (2002). Chem Phys Lett.

[6] Zheng L X, O'Connell M J, Doorn S K, Liao X Z, Zhao Y H, Akhadov E A, Hoffbauer M A, Roop B J, Jia Q X, Dye R C (2004). Nat Mater.

[7] Hong B H, Lee J Y, Beetz T, Zhu Y, Kim P, Kim K S (2005). J Am Chem Soc.

[8] Hata K, Futaba D N, Mizuno K, Namai T, Yumura M, Iijima S (2004). Science.

[9] Inoue Y, Kakihata K, Hirono Y, Horie T, Ishida A, Mimura H (2008). Appl Phys Lett.

[10] Patole S P, Alegaonkar P S, Shin H-C, Yoo J-B (2008). J Phys D: Appl Phys.

[11] Ivanov I, Puretzky A, Eres G, Wang H, Pan Z, Cui H, Jin R, Howe J, Geohegan D B (2006). Appl Phys Lett.

[12] Chakrabarti S, Nagasaka T, Yoshikawa Y, Pan L, Nakayama Y (2006). Jpn J Appl Phys.

[13] Hart A J, Slocum A H (2006). J Phys Chem B.

[14] Yun Y H, Shanov V, Tu Y, Subramaniam S, Schulz M J (2006). J Phys Chem B.

[15] Zhong G, Iwasaki T, Robertson J, Kawarada H (2007). J Phys Chem B.

[16] Yang Z, Chen X, Nie H, Zhang K, Li W, Yi B, Xu L (2008). Nanotechnology.

[17] Eres G, Puretzky A A, Geohegan D B, Cui H (2004). Appl Phys Lett.

[18] Li Q W, Zhang X F, DePaula R F, Zheng L X, Zhao Y H, Stan L, Holesinger T G, Arendt P N, Peterson D E, Zhu Y T (2006). Adv Mater.

[19] Christen H M, Puretzky A A, Cui H, Belay K, Fleming P H, Geohegan D B, Lowndes D H (2004). Nano Lett.

[20] Pan Z W, Xie S S, Chang B H, Wang C Y, Lu L, Liu W, Zhou W Y, Li W Z, Qian L X (1998). Nature.

[21] Zhang X, Cao A, Wei B, Li Y, Wei J, Xu C, Wu D (2002). Chem Phys Lett.

[22] Xiong G-Y, Wang D Z, Ren Z F (2006). Carbon.

[23] Hierold C (2008). Advanced Micro & Nanosystems, Carbon Nanotube Devices.

[24] Popp A, Yilmazoglu O, Kaldirim O, Schneider J J (2009). Chem Commun.

[25] Joshi R, Engstler J, Houben L, Bar Sadan M, Weidenkaff A, Mandaliev P, Issanin A, Schneider J J (2010). ChemCatChem.

[26] Zhang C, Pisana S, Wirth C T, Parvez A, Ducati C, Hofmann S, Robertson J (2008). Diamond Relat Mater.

[27] Amadou J, Bégin D, Nguyen P, Tessonnier J P, Dintzer T, Vanhaecke E, Ledoux M-J, Pham-Huu C (2006). Carbon.

[28] Janowska I, Winé G, Ledoux M-J, Pham-Huu C (2007). J Mol Catal A.

[29] Ducati C, Alexandrou I, Chhowalla M, Amaratunga G A J, Robertson J (2002). J Appl Phys.

[30] Hofmann S, Csányi G, Ferrari A C, Payne M C, Robertson J (2005). Phys Rev Lett.

[31] Fan S, Chapline M G, Franklin N R, Tombler T W, Cassell A M, Dai H J (1999). Science.

[32] Baker R T K (1989). Carbon.

[33] Li W Z, Xie S S, Qian L X, Chang B H, Zou B S, Zhou W Y, Zhao R A, Wang G (1996). Science.

[34] Andrews R, Jacques D, Rao A M, Derbyshire F, Qian D, Fan X, Dickey E C, Chen J (1999). Chem Phys Lett.

[35] Cheung C L, Kurtz A, Park H, Lieber C M (2002). J Phys Chem B.

[36] Joshi R, Schneider J J, Yilmazoglu O, Pavlidis D (2010). J Mater Chem.

[37] Tempel H, Joshi R, Schneider J J (2010). Mater Chem Phys.

[38] Lee C J, Park J, Huh Y, Lee J Y (2001). Chem Phys Lett.

[39] Fan S, Chapline M G, Franklin N R, Tombler T W, Cassell A M, Dai H J (1999). Science.

[40] Cheung C L, Kurtz A, Park H, Lieber C M (2002). J Phys Chem B.

[41] Wang H, Xu Z, Eres G (2006). Appl Phys Lett.

[42] Wei B, Vajtai R, Choi Y Y, Ajayan P M, Zhu H, Xu C, Wu D (2002). Nano Lett.

[43] Bendiab N, Almairac R, Sauvajol J-L, Rols S, Elkaim E (2003). J Appl Phys.

[44] Wang B N, Bennett R D, Verploegen E, Hart A J, Cohen R E (2007). J Phys Chem C.

[45] Wang B N, Bennett R D, Verploegen E, Hart A J, Cohen R E (2007). J Phys Chem C.

[46] Burian A, Dore J C, Hannon A C, Honkimaki V (2005). J Alloys Compd.

[47] Hough L A, Islam M F, Hammouda B, Yodh A G, Heiney P A (2006). Nano Lett.

[48] Guinier A (1994). X-ray diffraction in crystals, imperfect crystals, and amorphous bodies.

[49] Engel M, Sühn B, Schneider J J, Cornelius T, Naumann M (2009). Appl Phys A: Mater Sci Process.

[50] Rosenfeld Y (1990). Phys Rev A.

[51] Xie S S, Li W Z, Pan Z W, Chang B H, Sun L F (1999). Eur Phys J D.

[52] Kim N S, Lee Y T, Park J, Han J B, Choi Y S, Choi S Y, Choo J, Lee G H (2003). J Phys Chem B.

[53] Mahanandia P, Vishwakarma P N, Nanda K K, Prasad V, Barai K, Mondal A K, Sarangi S, Dey G K, Subramanyam S V (2008). Solid State Commun.

[54] Mahanandia P, Nanda K K (2008). Nanotechnology.

[55] Mahanandia P, Arya V P, Nanda K K, Bhotla P V, Subramanyam S V (2009). Mater Sci Eng, B.

